# Tranexamic acid may benefit patients undergoing total hip/knee arthroplasty because of haemophilia

**DOI:** 10.1186/s12891-019-2767-x

**Published:** 2019-09-04

**Authors:** Ze Yu Huang, Qiang Huang, Han Jiang Zeng, Jun Ma, Bin Shen, Zong Ke Zhou, Fu Xing Pei

**Affiliations:** 1 Department of Orthopedic Surgery, West China Hospital, West China Medical School, SiChuan University, 37# Wainan GuoXue Road, ChengDu, SiChuan Province People’s Republic of China; 2 Department of Radiology, West China Hospital, West China Medical School, SiChuan University, ChengDu, SiChuan Province People’s Republic of China

**Keywords:** Tranexamic acid, Total joint arthroplasty, Hip, Knee, Blood loss, Transfusion

## Abstract

**Background:**

The lower limb joints, including hip and knee, are the most commonly involved joints in haemophilic arthropathy. With a higher risk of transfusion, total hip and knee arthroplasty (THA and TKA) are still the first choice after failure of conservative treatment. In the present study, we aimed to analyze clinical outcomes and complications rate after total joint arthroplasty of the lower limbs using tranexamic acid (TXA) or not.

**Methods:**

Thirty-four patients with haemophilia A undergoing 24 TKA and 18 THA were evaluated in this retrospective study (No. 201302009). Based on using TXA or not, they were divided into either TXA (12 knees and 10 hips) or Non-TXA groups (12 knees and 8 hips). Total blood loss, intraoperative blood loss, total amount of FVIII usage, range of motion, inflammatory biomarkers, joint function, pain status, complication rate and patient satisfaction were assessed and compared at a mean follow-up of 68 months.

**Results:**

Usage of TXA can decrease not only the perioperative blood loss (*p* = 0.001), transfusion rate (*p* = 0.017) and supplemental amount of FVIII (*p* < 0.001) but also swelling ratio, surgical joint pain. Moreover, compared with non-TXA group, the patients in TXA group had a lower level of inflammatory biomarkers and better joint function.

**Conclusion:**

The hemophiliacs treated with TXA had less perioperative blood loss, hidden blood loss, transfusion rate, a lower ratio of postoperative knee swelling, less postoperative joint pain, lower levels of inflammatory biomarkers and better joint function. Further studies need performing to assess the long-term effects of TXA in these patients.

## Background

Haemophilia, recessive X-linked coagulation disorders, is caused by a deficiency of clotting factor VIII (FVIII) (haemophilia A) or FIX (haemophilia B) [1]. Its most common clinical manifestation is spontaneous bleeding in the musculoskeletal system [2, 3]. In severe cases, haemophilic arthropathy can attribute to blood-induced synovitis and cartilage damage caused by repetitive intra-articular hemorrhages. The most commonly involved joints are the lower limb joints, such as hip and knee and patients typically presented at a relatively young age in the form of a painful joint with a restricted range of motion (ROM), and functional decline [4, 5]. It has been confirmed that total hip and knee arthroplasty (THA and TKA) are of great benefit for pain relief, functional status restoration and subsequent bleeding episode decrease [6, 7]. Nowadays, it is the common practice for the surgeons to apply the total joint arthroplasty (TJA) in adult hemophiliacs displaying symptoms of advanced symptomatic arthrosis [8].

It is widely acknowledged that perioperative blood loss is one of the major complications following lower extremity TJA [9–11]. Additionally, hemophiliacs would be more likely to have a higher perioperative blood loss as a consequence of a decrease of the intrinsic clotting ability. That haemophilia is an independent risk factor for postoperative blood loss and transfusion has been demonstrated in several studies [12, 13].

Tranexamic acid (TXA) is an antifibrinolytic agent that blocks lysine binding sites on plasminogen, thereby inhibiting the formation of plasmin [9–11]. Therefore, TXA is believed to promote retention of intraoperative wound blood clots and reduce bleeding, confirmed by many studies [9, 11, 14]. However, whether TXA can benefit patients undergoing lower extremity TJA because of haemophilia still remains unknown. Since 2012, our center has widely used TXA perioperatively in patients undergoing lower extremity TJA. We take this study to address the following study questions: (1) How much can TXA usage minimize the perioperative blood loss and related parameters, such as transfusion rate associated with the patients treated with TJA for haemophilia; (2) Are there any other benefits hemophiliacs can gain from TXA usage? (3) Is it safe to use TXA in hemophiliacs?

## Methods

### Study sample

This retrospective study, based on data collected in our prospective database and approved by the Institutional Review Board of our institution, was performed at our center from January 2008 to August 2017 (No. 201302009). Inclusion criteria included lower extremity TJA (TKA and THA) secondary to haemophilia. Based on whether they were administered with TXA, patients were spontaneously divided into TXA group or non-TXA group. The class of severity for the patient was determined by their level of FVIII as follows: (1) mild:> 5- < 40%; (2) moderate: 1–5%; (3) severe: < 1%.

### Hematological management

Preoperatively, we assessed factor activity and screened for antibodies to determine pre- and perioperative factor substitution. Two thousand international units (IU) clotting factor VIII (FVIII) was administered to the patients on the day preoperatively. The activity of FVIII was monitored pre, post 1 h, post 2 h and post 4 h. Then the doctors would estimate how much of the FVIII would be needed during the perioperative period. The target factor activity level of the operative day was kept over 90%. In the following 3 days, the target factor activity level was over 80%. From the postoperative day 4 to day 7, the target level was over 50% and over 30% from the postoperative day 8 to day 14.

### Surgical procedure

One surgical team consisting of 2 senior orthopaedics surgeons performed all TJAs. The surgeons applied a standard posterolateral approach as previously described [10] for the patients undergoing THA, using the cementless prostheses (DePuy PINNACLE+CORAIL). For patients undergoing TKA, a standard medial parapatellar approach and a measured resection technique were used as previously described [9, 11], the prosthesis was a cemented total knee system (DePuy Sigma PFC). For patients in the TXA group, TXA was administered as described before [11]. Briefly, all patients in the TXA group received intravenous TXA 5 to 10 min before the skin incision (20 mg/kg) and 3, 6, 12, and 24 h later (10 mg/kg) along with 1 g of topical TXA in 50 mL of normal saline solution. Tourniquet was used in all patients undergoing TKA. The surgeons applied the same modern perioperative pain control, clinical and rehabilitation protocols in all patients as previously described [9–11]. Considering the characteristic of haemophilia patient, only mechanical methods including compression elastic stockings and intermittent pneumatic compression were used for prophylaxis.

### Outcome measurements

We collected data on patient demographics, medical history, concomitant medications, the length of hospital stays and complications, along with a complete blood-cell count, hepatic function, blood creatinine level, blood urea nitrogen level, prothrombin time, activated thromboplastin time and platelet count during the inpatient hospitalization in 1 week and 1, 3, and 6 months postoperatively. The complete blood-cell count, levels of inflammatory markers (C-reactive protein [CRP] and interleukin-6 [IL-6]) were tested on postoperative days 1, 2, 3, and 5 (POD 1, 2, 3 and 5). A doctor and a nurse made home visits to collect the study blood samples whenever necessary after discharge. We evaluated deep vein thrombosis (DVT) at the time of discharge and at 1, 3 and 6-month for follow-up whenever DVT was clinically suspected. Pulmonary embolism (PE) was diagnosed on the basis of clinical symptoms and an enhanced chest computer tomography (CT) scan.

We compared the total blood loss (calculated using the modified Gross Formula) [15], hidden blood loss (defined and calculated using Sehat’s formula [16] that subtracts the total measured blood loss from total blood loss), maximum decline (defined as the difference between the perioperative hemoglobin (Hb) level drawn closest to the time of the surgery and the minimal Hb level drawn postoperatively during the hospitalization or prior to any blood transfusion), total IU of FVIII usage, transfusion rate, CRP and IL-6 (POD 1, 2, 3 and 5) swelling ratio, the length of hospital stays, patient satisfaction, perioperative visual analog scale (VAS), (DVT) events, pulmonary embolism (PE) events and other complications between the two groups. The circumferences of hip or knee were measured as described by previous studies [9, 14, 17]. The swelling ratio was defined as the circumference of the operative limb divided by the circumference of the contralateral limb. After discharge, patients would be followed at 1 month, 6 months and every year postoperatively in clinic. The ROM (flexion/extension, internal/external rotation, adduction/abduction, degree), Harris hip score (HHS) and the Knee Society’s Knee Score (KSS) were recorded at each follow-up. All patients received radiographic assessment and examination for signs of implant migration or loosening, fixation of components and osteolysis. A component was considered loose if sequential radiographic series demonstrated macromotion, gross subsidence or progressive radiolucency of > 2 mm at interfaces [18]. All patients completed a satisfaction questionnaire regarding the outcomes of the surgery at the time of discharge and each follow-up time points. Satisfaction was rated on a 7-point scale, ranging from extremely dissatisfied to extremely satisfied.

### Statistical analysis

All data management and statistical analysis were performed with SPSS version 18.0 software (SPSS Inc., Chicago, IL, USA). Independent t-tests were used for continuous variables such as maximum decline, BMI, age, etc. Pearson chi-square test or Fisher exact test was used to analyze the categorical variables. The level of significance was set at *p* < 0.05.

## Results

### Patient demographics

Among the total 34 patients (24 knees and 18 hips), 14 patients underwent unilateral TKA, 12 patients unilateral THA, 5 patients simultaneously bilateral TKA and 3 patients underwent simultaneously bilateral THA. Preoperative patient demographics showed no statistically significant differences between the two groups in terms of age, BMI, disease severity, Hb, Hct, platelet count, international normalized ratio (INR), APTT, preoperative FVIII activity, VAS score or FVIII inhibitor (Table 1). No significantly statistical difference exists in duration of surgery between the groups (Table 2). The average of follow-up time was 68 ± 28 months.

**Table 1:** Preoperative Demographics

Demographics	Non-TXA Group (*N* = 18)	TXA Group (*N* = 16)	*p* Value
Age^a^ (yr)	37.2 ± 11.8	38.9 ± 11.3	0.681
Sex^b^ (male/female)	18/0	16/0	1.000
BMI^a^ (kg/m^2^)	23.2 ± 5.8	23.4 ± 2.4	0.941
Severity^b^			0.597
mild	2	1	
moderate	6	8	
severe	10	7	
Joints^b^			0.957
Unilateral TKA	8	6	
Unilateral THA	6	6	
Simultaneous TKA	2	3	
Simultaneous THA	2	1	
Hb^a^ (g/L)	129.2 ± 12.3	128.0 ± 10.0	0.765
Hct^a^ (%)	0.40 ± 0.04	0.40 ± 0.03	0.819
Platelet count^a^ (× 10^9^/L)	138 ± 44	149 **±** 58	0.561
INR^a^	0.99 ± 0.97	1.00 ± 0.10	0.891
APTT^a^ (sec)	92.3 ± 26.1	92.4 ± 21.5	0.986
Preoperative FVIII activity^a^ (%)	2.13 ± 1.12	2.27 ± 1.06	0.72
VAS score^a^ (0–10, 10 worst)	4.5 ± 1.10	4.75 ± 1.48	0.578
FVIII inhibitor^b^ (negative/positive)	5/13	6/10	0.717
Knee	*N* = 12	*N* = 12	
ROM^a^ (Flexion/Extension) (°)	52 ± 23	49 ± 20	0.779
KSS score^a^	33 ± 20	30 ± 19	0.679
Hip	*N* = 10	*N* = 8	
ROM Flexion/Extension^a^ (°)	59 ± 21	62 ± 20	0.768
ROM Internal−/External-Rota^a^ (°)	17 ± 4	15 ± 8	1.000
ROM Adduction/Abduction^a^ (°)	28 ± 20	23 ± 18	0.555
HHS score^a^	35 ± 15	31 ± 14	0.543

Abbreviations: *TXA* tranexamic acid; *BMI* body mass index; *TKA* total knee arthroplasty *THA* total hip arthroplasty; *Hb* hemoglobin; *Hct* hematocrit; *INR* international normalized ratio; *APTT* activated partial thromboplastin time; *VAS* visual analog scale; *ROM* range of motion; *KSS* Knee Society’s Knee Score; *HHS* Harris Hip Score

^a^ The values are given as mean ± standard deviation

^b^ The values are given as number of patients

**Table 2:** Preoperative Demographics

Demographics	Non-TXA Group (*N* = 18)	TXA Group (*N* = 16)	*p* Value
Duration of surgery (min)^a^	89.1 ± 30.1	87.4 ± 30.4	0.873
Total blood loss (mL)^a^	1464 ± 480	935 ± 394	0.001
Intraoperative blood loss (mL)^a^	397 ± 183	264 ± 69	0.009
Drainage volume (mL)^a^	354 ± 119	236 ± 91	0.003
Hidden blood loss (mL)^a^	713 ± 474	435 ± 277	0.043
Amount of FVIII usage (IU)^a^	42,125 ± 1965	36,328 ± 4858	< 0.001
Transfusion rate (%)^b^	61%	19%	0.017
Transfusion amount (RBC suspension) per patient (mL)^a^	178 ± 166	38 ± 81	0.004
Transfusion amount (plasma) per patient (mL)^a^	56 ± 92	13 ± 50	0.106
Swelling ratio (Knee) (%)^a^			
POD1	113.0 ± 3.8	107.0 ± 3.5	0.001
POD2	116.2 ± 4.7	108.8 ± 3.6	< 0.001
POD3	116.9 ± 4.9	109.5 ± 3.4	< 0.001
POD5	116.2 ± 4.2	106.6 ± 2.8	< 0.001
Swelling ratio (Hip) (%)^a^			
POD1	115.6 ± 6.4	111.0 ± 4.8	0.099
POD2	118.7 ± 3.6	113.1 ± 4.4	0.012
POD3	118.1 ± 4.3	113.8 ± 4.6	0.058
POD5	115.1 ± 4.3	110.1 ± 2.9	0.010
Maximum Hb change (g/L)^a^	32 ± 6	22 ± 7	< 0.001
Length of hospital stay^a^	20 ± 6	14 ± 4	< 0.001

Abbreviations: *TXA* tranexamic acid; *RBC* red blood cell; *POD1* postoperative day 1; *POD2* postoperative day 2; *POD3* postoperative day 3; postoperative day 5; *Hb* hemoglobin

^a^ The values are given as mean ± standard deviation

^b^ The values are given a percentage

### Blood loss

Significant differences were detected between groups for all measured variables (Table 2). The drainage volume in TXA group (236 ± 91 mL) was significantly lower than that in Non-TXA group (354 ± 119 mL, *p* = 0.003). Also, the hidden blood loss was significantly lower in TXA group compared to Non-TXA group (435 ± 277 mL VS. 713 ± 74 mL, *p* = 0.043) (Table 2). Significantly higher maximum Hb change was observed in Non-TXA group (32 ± 6 g/L VS. 22 ± 7 g/L, *p* < 0.001). Eleven patients in Non-TXA group received transfusion while only three patients in TXA group needed transfusion during the in-patient period. TXA group had a significantly lower transfusion rate and a less transfusion amount per patient than Non-TXA group (42,125 ± 1965 IU VS. 36,328 ± 4858 IU, *p* < 0.001) (Table 2).

### Postoperative general assessment

**Fig. 1:**
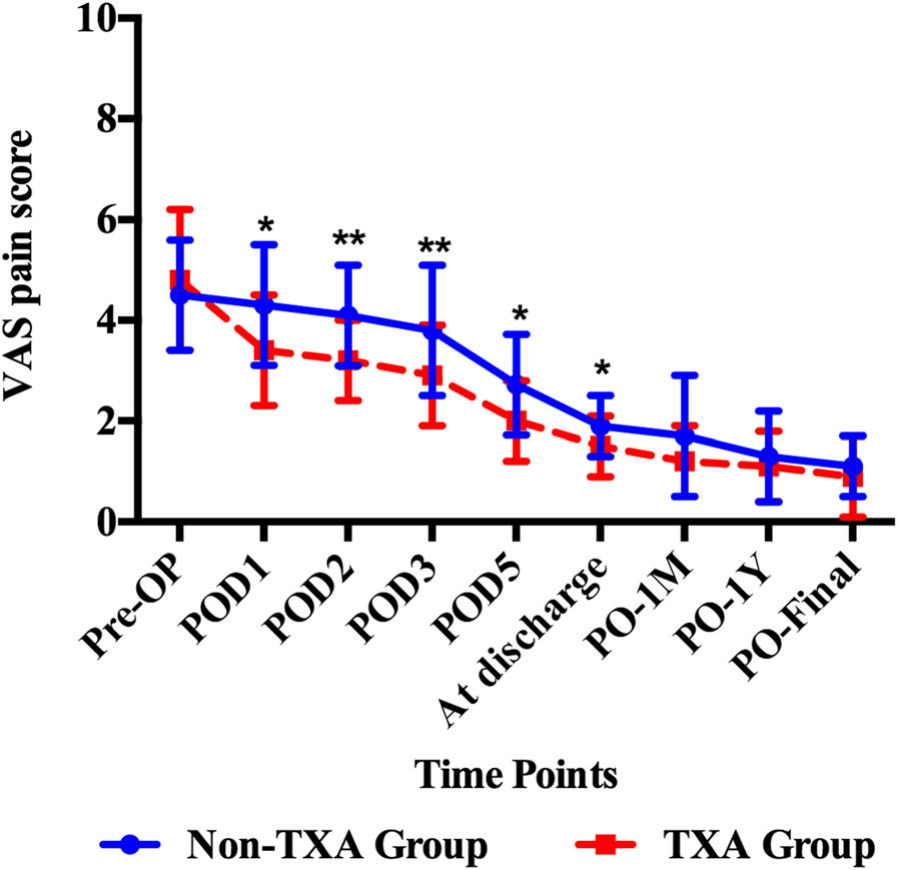
VAS pain was reduced in TXA compare with Non-TXA use. The mean (standard deviation, SD) longitudinal VAS pain scores in both groups. Pre-OP = preoperative on morning of surgery, POD = postoperative days 1 through 5 (POD1, POD2, POD3, POD5); PO = postoperative months 1 through final follow-up (PO-1 M, PO-1Y, PO-Final). The asterisks indicate *p* values that were significantly different between two groups (calculated by independent t-tests, ** = *p* < 0.01, * = *p* < 0.05) (Error bar stands for the SD)

Pain of the operated joint decreased continually postoperatively. Patients in TXA group had less postoperative pain than preoperatively at each time point, with the significant differences between the two groups persisting through day at discharge (Fig. 1). The knee swelling ratio was significantly better in TXA group on POD 1, 2, 3 and 5 (*p* = 0.001, *p* < 0.001, *p* < 0.001, and *p* < 0.001). The hip swelling ratio was significantly better in TXA group on POD 2 and 5 (*p* = 0.012, and *p* = 0.010). Both groups had a substantial improvement in the range of motion at the time of discharge (Table 3). As to the Knee ROM, patients in TXA group tended to be significantly better than those from non-TXA group at the time of discharge, 1-year follow-up and final follow-up (*p* = 0.004, *p* = 0.004 and *p* = 0.013, respectively). The average KSS score increased from 33 ± 20 to 83.0 ± 5.2 in the non-TXA group and from 30 ± 19 to 88.2 ± 5.7 in the TXA group. KSS score was significantly higher in the TXA group at discharge, 1-year follow-up and final follow-up compared to the non-TXA group (*p* = 0.023, *p* = 0.026 and *p* = 0.028, respectively). As to the Hip, patients in TXA group had a better ROM in terms of flexion/extension (*p* = 0.027, *p* = 0.006 and *p* = 0.022, respectively) and internal/external-rotation (*p* < 0.001, *p* < 0.001 and *p* < 0.001, respectively) at the time of discharge, 1-year follow-up and final follow-up (Table 3). The average HHS score increased from 35 ± 15 to 80.5 ± 7.7 in the non-TXA group and from 31 ± 14 to 89.1 ± 4.5 in the TXA group. The HHS score was significantly better in the TXA group at the time of discharge, 1-year follow-up and final follow-up (*p* = 0.036, *p* = 0.020 and *p* = 0.017, respectively).

**Table 3:** Clinical outcomes

Demographics	Mean and Standard Deviation		*p* Value
	Non-TXA Group (*N* = 18)	TXA Group (*N* = 16)	
Knee	*N* = 12	*N* = 12	
ROM (Flexion/Extension) at discharge (°)	92.9 ± 5.0	100.8 ± 6.7	0.004
ROM (Flexion/Extension) at 1-year follow-up (°)	92.5 ± 4.0	100.0 ± 7.0	0.004
ROM (Flexion/Extension) at final follow-up (°)	95.0 ± 3.0	101.3 ± 7.1	0.013
KSS score at discharge	79.2 ± 6.7	86.2 ± 5.4	0.023
KSS score at 1-year follow-up	82.2 ± 5.0	88.1 ± 5.5	0.026
KSS score at final follow-up	83.0 ± 5.2	88.2 ± 5.7	0.028
Hip	*N* = 10	*N* = 8	
ROM (Flexion/Extension) at discharge (°)	102.5 ± 8.2	113.1 ± 9.6	0.027
ROM (Flexion/Extension) at 1-year follow-up (°)	105.5 ± 4.4	113.8 ± 5.8	0.006
ROM (Flexion/Extension) at final follow-up (°)	104.0 ± 5.2	111.3 ± 6.4	0.022
ROM (Internal−/External-Rota) at discharge (°)	39.0 ± 7.4	61.9 ± 6.5	< 0.001
ROM (Internal−/External-Rota) at 1-year follow-up (°)	39.0 ± 11.0	68.8 ± 9.9	< 0.001
ROM (Internal−/External-Rota) at final follow-up (°)	40.5 ± 6.0	65.6 ± 7.8	< 0.001
ROM (Adduction/Abduction) at discharge (°)	48.0 ± 12.3	62.5 ± 15.8	0.053
ROM (Adduction/Abduction) at 1-year follow-up (°)	50.5 ± 9.7	55.0 ± 22.4	0.622
ROM (Adduction/Abduction) at final follow-up (°)	50.5 ± 10.1	55.0 ± 21.8	0.658
HHS score at discharge (°)	76.5 ± 10.1	86.1 ± 4.6	0.036
HHS score at 1-year follow-up (°)	78.8 ± 8.8	88.1 ± 5.5	0.020
HHS score at final follow-up (°)	80.5 ± 7.7	89.1 ± 4.5	0.017

Abbreviations: *TXA* tranexamic acid; *ROM* range of motion; *KSS* Knee Society’s Knee Score; *HHS* Harris Hip Score

The values are given as mean ± standard deviation

**Fig. 2:**
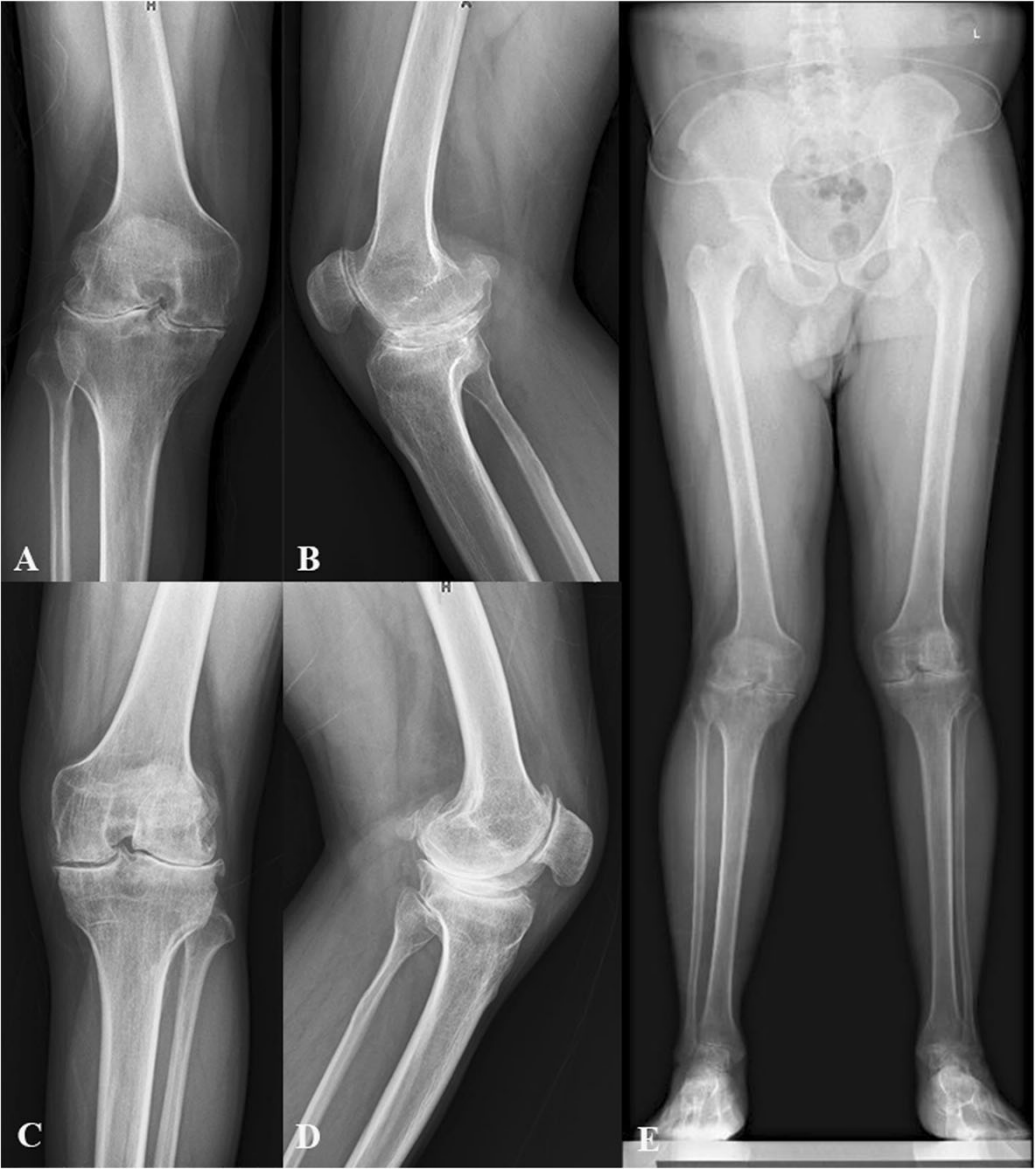
Preoperative X-ray of haemophilic arthropathy of a patient seeking for bilateral TKA. **a** Anteroposterior (AP) view of right knee; **b** Lateral view of right knee; **c** Anteroposterior (AP) view of left knee; **d** Lateral view of left knee; **e** Full-length standing AP view

**Fig. 3:**
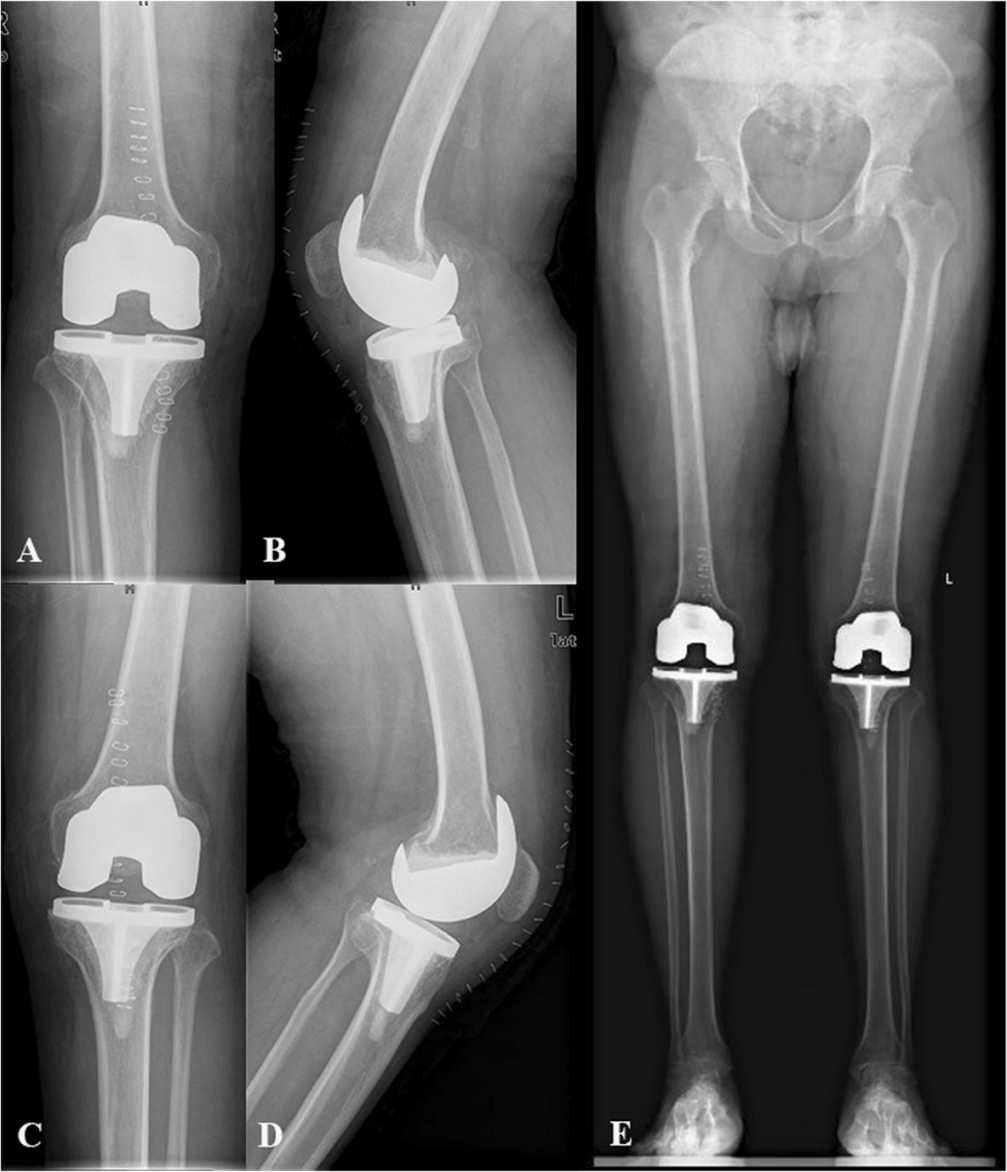
Postoperative X-ray of simultaneous bilateral TKA. **a** AP view of right knee; **b** Lateral view of right knee; **c** Anteroposterior (AP) view of left knee; **d** Lateral view of left knee; **e** Full-length standing AP view

**Fig. 4:**
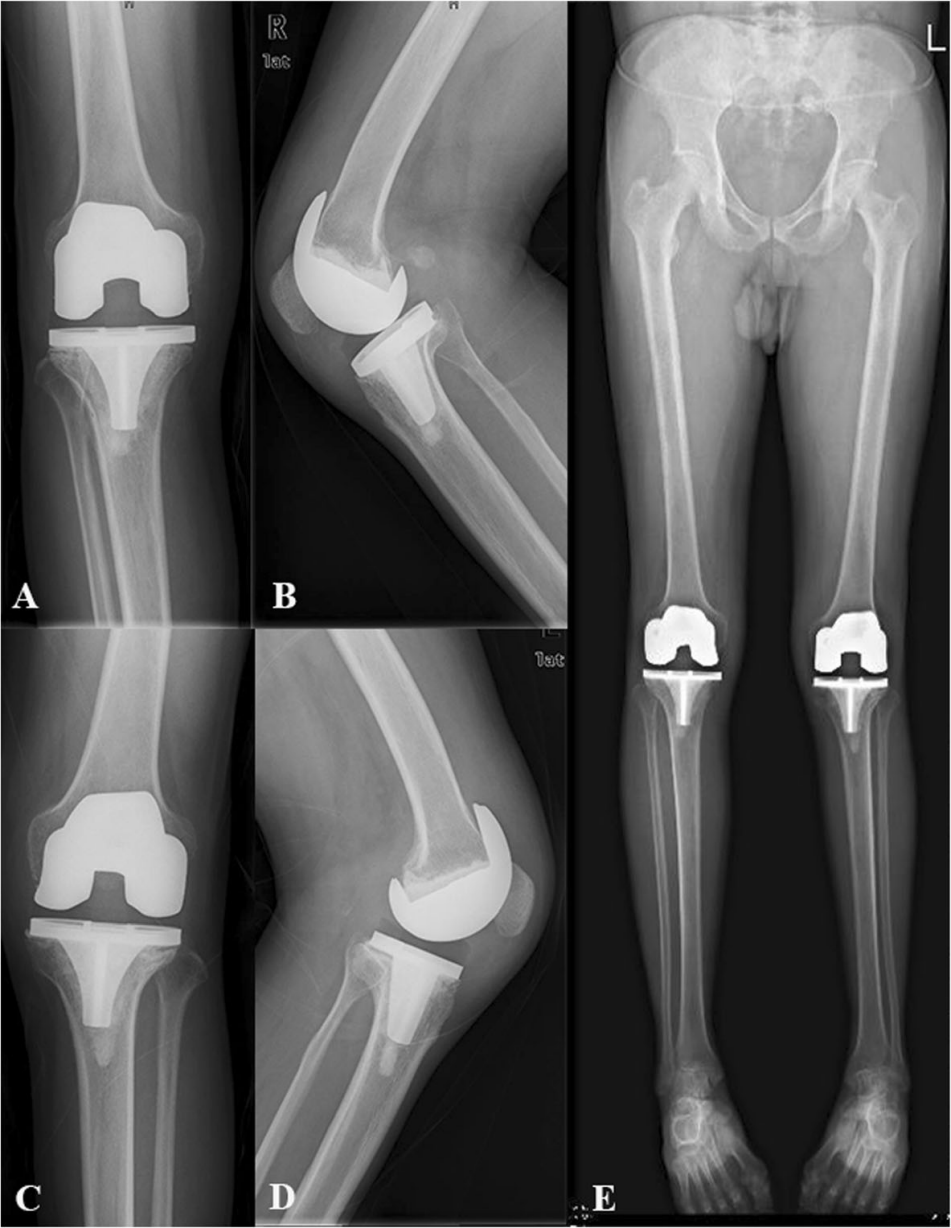
One year follow-up X-ray of simultaneous bilateral TKA. **a** AP view of right knee; **b** Lateral view of right knee; **c** Anteroposterior (AP) view of left knee; **d** Lateral view of left knee; **e** Full-length standing AP view. (No progressive radiolucent line > 2 mm in width)

**Fig. 5:**
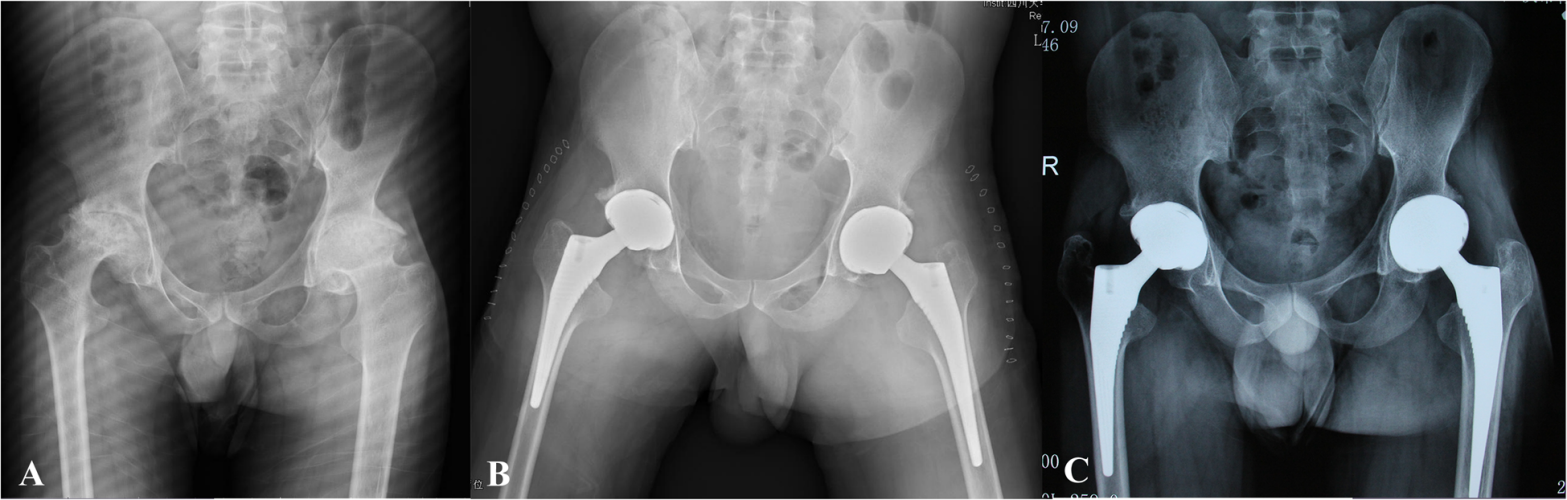
Preoperative and postoperative X-ray of a patient seeking for bilateral THA because of haemophilia A. **a** Preoperative anteroposterior (AP) view of pelvis; **b** Posoperative AP view of pelvis; **c** One year follow-up AP view of pelvis (No progressive radiolucent line > 2 mm in width)

Serious follow-up X-ray illustrated remaining of the good component position in all patients (Figs. 2, 3, 4 and 5). There was no progressive radiolucent line > 2 mm in width.

### Inflammatory markers

**Fig. 6:**
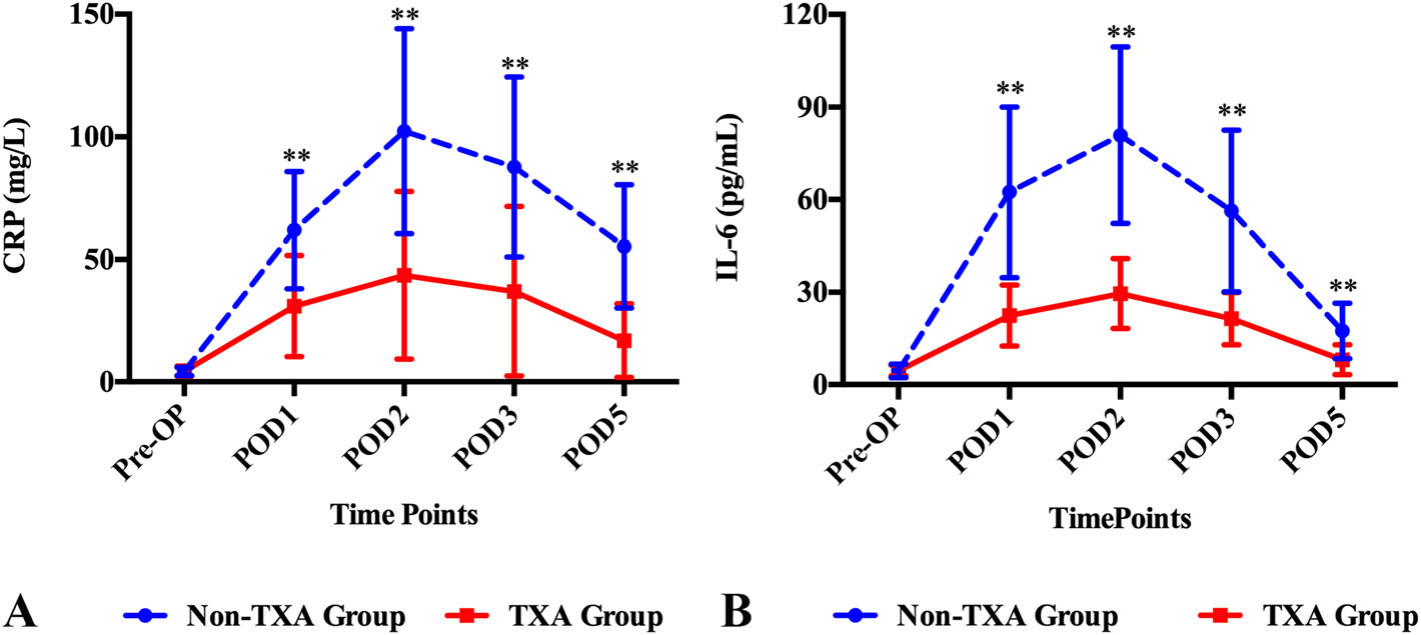
Perioperative inflammation was reduced by TXA compared with Non-TXA use. The mean (SD) of serum concentrations of inflammatory biomarkers CRP (**a**) and IL-6 (**b**) during the perioperative first 5 days after TJA. Pre-OP = preoperative on morning of surgery, POD = postoperative days 1 through 5 (POD1, POD2, POD3, POD5). The asterisks indicate *p* values that were significantly different between two groups (calculated by independent t-tests, ** = *p* < 0.01, * = *p* < 0.05) (Error bar stands for the SD)

The CRP and IL-6 levels increased postoperatively in all patients (Fig. 6). The CRP level, reaching its peak on postoperative day 2 in both groups, was significantly lower in TXA group compared with non-TXA group on postoperative days 1, 2, 3 and 5 (*p* < 0.001 for all). The IL-6 level also peaked on the postoperative day 2 in both groups and similar to the CRP level, significantly lower in TXA group compared with non-TXA group on postoperative days 1, 2, 3 and 5 (*p* < 0.001, *p* < 0.001, *p* < 0.001 and *p* = 0.001, respectively).

### Complications

Neither DVT nor PE occurred in any patients. Two knees in the non-TXA group and one knee in the TXA group developed knee stiffness within 1 year postoperatively. Surgical releasing was performed in all three knees. No wound secretion was observed in TXA group during the entire follow-up. Blistering was reported in 4 patients in the non-TXA group. Hemorrhage was reported in one patient from the TXA group at postoperative month 3 because of too much activity. The patient was treated with intravenous FVIII factor. No periprosthetic fracture and periprosthetic joint infection (PJI) was reported in all patients from both groups during the entire follow-up period.

### Patients’ satisfaction

In the final follow-up, 100% of the patients from both groups were satisfied. There was no significant difference between two groups in terms of satisfaction at postoperative time points. All patients agreed to undergo the operation again.

## Discussion

The most important finding of the present study was that usage of TXA can decrease not only the perioperative blood loss, transfusion rate and supplemental amount of FVIII but also swelling ratio and surgical joint pain. Moreover, compared with non-TXA group, the patients in TXA group had a lower level of inflammatory biomarkers and better joint function.

The perioperative blood loss in primary TJA includes overt blood loss, composed of intraoperative blood loss and postoperative drainage, and hidden blood loss, such as extravasation into the tissue, residual blood in the joint, and loss due to hemolysis, accounts for even 50% of the total blood loss [19]. According to our previous comparable studies available in non-haemophilic patients [9–11, 14], using combination of intravenous and topical application of TXA could gain less total blood loss, less intraoperative blood loss, maximum Hb decline and less drainage. In the current study, we found similar benefits of using TXA in haemophilic patients. What is more, we also found that using TXA could significantly reduce the total amount of FVIII factors during the perioperative period. This extra finding was also suggested by studies [20–22] focusing on the TXA as adjunct therapy to daily treatment of haemophilia A patients with inhibitors. The possible mechanism is that TXA can stabilize clot, improving haemostatic effect in patients with haemophilia.

In the current study, we also found that patients using TXA tend to have a significant lower level of inflammatory biomarkers, such as CRP and IL-6. There are several possible reasons underlying the anti-inflammatory effects observed in TXA group. Firstly, previous studies showed that TXA could attenuate inflammatory responses through blockade of fibrinolysis [23–25]. Secondly and most importantly, TXA reduced total blood loss, translating into reduced total surgical trauma [17] since postoperative blood loss and pain were shown to positively synergize with postoperative inflammation and surgical trauma as demonstrated by CRP, IL-6 and IL-1 levels [17, 26], which might also explain why there was a lower joint swelling ratio observed in the TXA group during the postoperative days. Holem et al. [27] have demonstrated that joint swelling after TJA is mainly due to intra-articular bleeding and inflammation of the periarticular tissues. Since TXA can decrease not only blood loss but also local inflammation [14, 28, 29], it would not be surprising to find that patients in TXA group had a significantly lower swelling ratio compared to those from non-TXA group.

In most patients with haemophilia, arthrofibrosis accompanies the joint destruction seen with the recurrent bleeding episodes within the joint [8, 13]. Patients often have significantly limited ROM of the joints. In our study, a significant increase in ROM and a reduction of the pain after TJA in haemophilic patients was observed in both groups. Furthermore, substantial improvements of joint function as determined by either KSS or HHS were demonstrated. The results were comparable with the follow-up results of haemophilic patients undergoing TJA reported by previous studies [30, 31]. In addition, we observed that patients in the TXA groups would have better clinical outcomes at in the early follow-up time points both measured by ROM and functional scales. There might be several reasons for this. For one thing, swelling and intra-articular bleeding have been reported to associate with decreased ROM and function especially in patients with bleeding disorders [13]. Because of using TXA, swelling and intra-articular bleeding were significantly decreased, thus the patients would have a better ROM and function. For another, patients in TXA group had less pain in their operated joints. With better pain control in those patients, they would be more likely to involve into the early rehabilitation. It has been previously reported that although ROM is significantly improved in patients with haemophilia, a substantial number of patients will require manipulation under anesthesia and even lysis of adhesions to gain or maintain a functional ROM [8, 32, 33]. However, we did not observe re-stiffness in our patients. That might be attributed to our emphasis on the early-reached required ROM in the rehabilitation program, that is to encourage patients to reach their maximum ROM during the first 48 h postoperatively before the contracture of the fibrous tissue around the surgical site and the previous rehabilitation is to maintain the maximum ROM.

Previous literatures have reported patients receiving TJA because of haemophilia had significant higher infection rates ranging from 8 to 16% [6, 8, 34]. In our study, no periprosthetic joint infection was found at the last follow-up time points, which we believe mainly lies in the relatively short follow-up. With a special emphasis on late PJI, Rodriguez-Merchan et al. reported an infection rate of 2.8% at early follow-up [34] and then a rate of 6.8% at a later follow-up [3]. Some other groups even reported that four of five knees with PJI had late infection, and the average time for revision was 12 ± 4 years. Thus, we have emphasized the possibility of PJI in these patients and asked them to do the follow-up at least once every year.

The main concern of using TXA in patients undergoing TJA is the possibility of increasing the risk of DVT and PE postoperatively. However, it has been previously reported that DVT rate in patients with haemophilia was considered very low due to impaired coagulation activity. Herman et al. [35] reported that among 29 orthopaedic surgeries in 22 patients without using pharmacological thrombo-prophylaxis, only three subclinical DVTs were detected by Doppler ultrasound. In our study, we did not use pharmacological thrombo-prophylaxis in either groups. We performed Doppler ultrasound at POD5, the time of discharge, 1, 3 and 6-month follow-up. No DVT events were reported in both groups. This finding was consistent with the outcome reported by previous studies that the rate of DVT was much lower than that in non-haemophilia patients. None of the patients required CT to rule out PE.

There are limitations to our study. First and foremost, it is the retrospective design nature. We included the patients with enough follow-up information and otherwise excluded to avoid potential selection. Secondly, we did not calculate the needed sample size before the study. A larger sample size might be needed to detect significance in assessment outcomes between the groups. Third longer follow-up was needed to assess the long-term effects of TXA. The strength of our study that all procedures were performed by a single team of surgeons at a single academic institution using modern perioperative management protocols.

## Conclusion

In conclusion, we found using TXA in patients undergoing lower extremity TJA because of haemophilia A could have multiple benefits, including a decrease in total perioperative blood loss, transfusion rate, amount of usage of FVIII, postoperative joint swelling and better function. Further prospective designed studies needed to be performed to assess the long-term effects of TXA in these certain groups of patients.

## Data Availability

The datasets used and analyzed during the current study are available from the corresponding authors on reasonable request.

## Abbreviations

CRP: C-reactive protein
CT: Computer tomography
DVT: Deep vein thrombosis
Hb: Hemoglobin
HHS: Harris Hip Score
IL-6: Interleukin-6
INR: International normalized ratio
IU: International units
KSS: Knee Society knee score
PE: Pulmonary embolism
PJI: Periprosthetic joint infection
POD: Postoperative days
ROM: Range of motion
THA: Total hip arthroplasty
TJA: Total joint arthroplasty
TKA: Total knee arthroplasty
TXA: Tranexamic acid
VAS: Visual analog scale
